# Industrial Artificial and Natural Fibers’ Cutting Mechanism—A Review

**DOI:** 10.3390/mi17050513

**Published:** 2026-04-23

**Authors:** Shanshan Hu, Mengmeng Ma, Zhiliang Wu, Yuyuan Huang, Qingrui Jiang, Chengji Yang

**Affiliations:** 1 College of Mechanical Engineering, Guangxi University, Nanning 530004, China; hsswhh@gxu.edu.cn (S.H.); 2311391087@st.gxu.edu.cn (M.M.); 2411391036@st.gxu.edu.cn (Y.H.); 2 Guangxi MESDA Engineering Machinery Co., Ltd., No.97 Yuanyi Road, Nanning 530000, China; wuzhiliang@mesda.com.cn

**Keywords:** artificial fiber, natural fibers, cutting mechanism, tool wear

## Abstract

Industrial synthetic and natural fibers play an indispensable role in modern manufacturing, aerospace, automotive, and textile engineering. However, the enhanced mechanical performance of advanced industrial fibers has introduced significant challenges in cutting processes, since brittle, high-tensile, and viscoelastic fibers exhibit totally different fracture behaviors from conventional solid materials. At present, the complex motion coupling mechanisms between fibers and cutting tools under free-form conditions are insufficient; there is no unified framework for understanding the mechanisms of fiber cutting; it is difficult to effectively link the microscopic fracture physics of different fiber types with their macroscopic cutting properties. Furthermore, research into the dynamic interaction between the cutting tool and the fiber, cross-scale cutting characteristics, and tool wear mechanisms has not been sufficiently systematic, and non-contact cutting methods have not yet been the subject of systematic study. Through a systematic review, this review identified three primary categories of difficult-to-cut industrial fibers and summarized the distinctions in their fundamental material properties. The static, kinematic, and dynamic characteristics of fiber cutting under both free and fixed forms were discussed. The fracture mechanisms of fibers under diverse loading scenarios were also systematically revealed. Furthermore, this review summarizes the effects of cutting tool wear characteristics, geometric parameters, and material types on cutting performance. Finally, non-contact methods for cutting fiber were listed. Based on the above analysis, three critical directions for future research were proposed to bridge the existing knowledge gaps in the literature. This review of the interdisciplinary interactions among mechanics, materials science, and textile engineering provides a theoretical foundation and research directions for achieving high efficiency and a long tool life during cutting industrial fibers.

## 1. Introduction

Since ancient times, natural and artificial fibers have been widely used in human life and industry. Life experience tells us that, compared with metals, cutting long fibers is relatively easy and does not seem to cause much trouble in manufacturing. However, thanks to advancements in modern textile and chemical technologies, the properties of both artificial and natural fibers are becoming increasingly excellent. Various modified fibers are now extensively used in industry, national defense, and medical care. For instance, synthetic industrial fibers are applied as cables [1] and automotive components [2], high-performance fibers are often used as reinforcements in composites [3,4,5] or in extreme environments [6,7] and natural industrial fibers serve as sustainable and eco-friendly alternatives to traditional synthetic materials in automotive [8], aerospace [9], construction [10], packaging [11], and textiles [12]. On the one hand, these advanced fibers offer better performance and reliability; on the other hand, they introduce significant challenges to manufacturing processes.

As highly deformable soft materials, fibers exhibit different behaviors depending on their material properties: they can be brittle (such as SiC fiber, glass fiber, and carbon fiber), tough (such as flax, hemp, ramie, and jute), or even viscoelastic (such as ultra-high-molecular-weight polyethylene fiber). These material characteristics make their cutting processes significantly different from the fracture mechanisms of solid materials, which is the primary factor influencing cutting from a microscopic perspective. Since this paper pays attention to the manufacturing challenges posed by industrial artificial and natural fibers in industrial applications, the interaction between the cutting tool and fiber is the second key influencing factor from a macroscopic perspective.

Aiming to establish a comprehensive understanding of existing knowledge on fiber cutting to enable the efficient production of fiber-based products while preserving their excellent physicochemical properties, this review therefore focuses on those difficult-to-cut fibers and their cutting processes that demand exceptionally high cutting quality. Those fibers that provide sufficient strength to bear the load in components are selected as the difficult-to-cut fibers, such as glass fiber, carbon fiber, flax, hemp, ramie, jute, and ultra-high-molecular-weight polyethylene (UHMWPE) fiber.

Due to variations in industrial production processes, fiber cutting typically occurs in two distinct forms. One is cutting fibers before they are manufactured into other products, where they predominantly exist in a natural or tensioned state with variable deformability; the other is cutting fibers after they are fabricated into fiber-reinforced composites, where the fibers are cut in a non-free state embedded within a matrix and pre-impregnated. Based on the aforementioned industrial production processes, there are various ways to convert long fibers into short fibers. As long as the desired result of cutting is achieved, they all fall within the scope of this discussion. Therefore, the following, under different names, can all be considered cutting processes in this paper: cutting, chopping, slashing, shearing, breaking, and cutting off.

This review covers the fibers’ material characteristics, the fibers’ breaking mechanics at the microscale, the fibers’ cutting and chopping mechanisms at the macroscale, and the manufacturing and processing technologies for fiber cutting at different scales. Moreover, the fiber breaking in composite materials was also discussed.

The key properties and areas of application of different fibers are shown in Table 1.

## 2. The Material Characteristics of Fibers

The distinct characteristics of materials result in entirely different cutting mechanisms. Therefore, it is essential to thoroughly understand the material properties to address the situations that arise during the cutting process. Currently, the commonly used fibers that are difficult to cut in industrial applications fall into three categories: brittle fibers, high tensile strength natural fibers, and viscoelastic fibers.

(1) Brittle fibers

Brittle fibers are a widely used type of material characterized by their low strain-to-failure, high stiffness, and minimal plastic deformation before fracture. This brittleness originates from their inherent molecular and microstructural architecture—typically featuring strong covalent or ionic bonding networks that resist dislocation motion, which is the opposite of the primary mechanism of plasticity. Consequently, these materials, including glass, carbon, silicon carbide (SiC), and various ceramic fibers, quickly fail once their elastic limit is exceeded. This mechanical performance of brittle fibers strives to avoid surface and internal flaws. For fiber cutting, it means reducing the unevenness of the fracture surfaces. According to Griffith’s theory of brittle fracture [15,16,17], microscopic surface flaws and internal voids are potential stress concentrations, drastically reducing the effective strength of the fiber far below its theoretical cohesive strength, which is called the “weakest-link” principle. This is the reason why brittle fibers exhibit large variability (scatter) in tensile strength [18].

(2) High tensile strength natural fibers

High tensile strength natural fibers are a critical class with high tensile strength, fracture toughness, fatigue resistance, and dimensional stability under mechanical or environmental stress. Among the most prominent examples are bast fibers such as flax, hemp, ramie, and jute, as well as leaf fibers such as sisal and pineapple. Their robust mechanical properties derive from their hierarchical microstructure and high cellulose content. The tensile strength is a co-function of several interrelated factors, including cellulose content, crystallinity, microfibril angle, fiber morphology, plant age, environmental growing conditions, and the specific extraction and post-processing methods employed. Among these, the cellulose content is particularly important, which consists of strong, stiff cellulose microfibrils acting as the primary load-bearing reinforcement. Ramie fiber stands out as possessing the highest tensile strength of over 1000 MPa among common natural bast fibers [19] with high cellulose content ranging from about 70% to over 90% [20,21]. Flax fiber is another top natural fiber with tensile strengths reported in the range of 800–1100 MPa [22,23]. Its high strength is also a consequence of its high cellulose content (around 60–80% [24,25,26]) and a favorable microfibril orientation that efficiently channels applied loads [27].

From the above analysis, the high tensile strength of these fibers creates difficulties in cutting them.

(3) Viscoelastic fibers

Viscoelastic fibers are a critical class of polymeric materials that exhibit both viscous (fluid-like) and elastic (solid-like) characteristics when subjected to deformation. This dual nature is affected by time-, temperature-, and strain-rate-dependent responses, which expand their applications from biomedical scaffolds to advanced composites and smart textiles. The mechanical behavior of viscoelastic fibers arises from their molecular architecture—specifically, the mobility, entanglement, and reversible physical crosslinks (e.g., hydrogen bonds, hydrophobic domains) among polymer chains. These structural features enable energy dissipation through internal friction while retaining the capacity for shape recovery, a typical sign of viscoelasticity.

**Figure 1:**
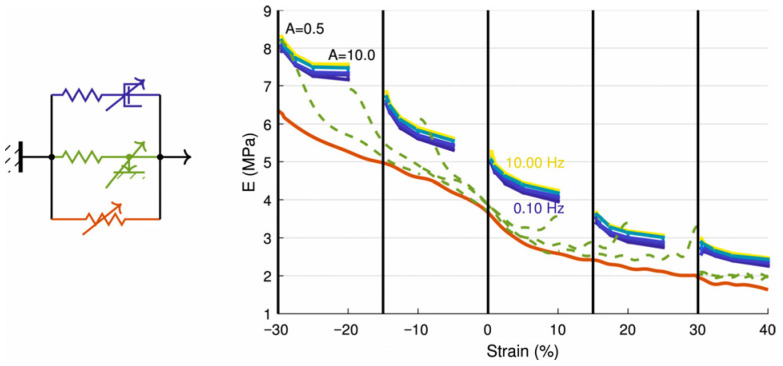
Unified non-parametric representation of hyperelastic (solid red), hysteretic relaxation (green, dashed), viscoelastic complex moduli (blue to yellow map) [36].

Rheological models such as the Maxwell and Kelvin–Voigt frameworks under different conditions are the basis for understanding the foundational mechanics of viscoelastic fibers [28,29]. These models were based on the combination of a spring and dashpot in series to describe the transient elasticity, stress relaxation, and irreversible flow of viscoelastic fluids. However, the Kelvin–Voigt solid does not have an instantaneous elastic response to simulate real solid features. Therefore, more sophisticated models, such as the Standard Linear Solid (SLS) [30,31] or Generalized Linear Viscoelastic Models [32,33], combine these elements to replicate the full spectrum of transient responses observed in fiber modeling. The aforementioned methods still solve problems within a linear framework. However, under complex loading conditions, particularly during cutting, fibers experience high stress and drastic load variations. It often exhibits nonlinear viscoelastic characteristics, in which the material’s stress–strain relationship is nonlinear. Consequently, a series of nonlinear viscoelastic models have been developed. Typical nonlinear models included the power-law model [34], the hyperbolic tangent model [35,36] (Figure 1), the Kaye–Bernstein (K-B) model [37,38], etc. Figure 1 illustrates hyperplastic, hysteretic and viscoelastic complex moduli (ratio between stress and strain variations) as a function of static operating strain [36], which provides the base knowledge on the dynamic strain under the effect of cutting. Among these, the power-law model assumed that the relationship between stress and strain followed a power-law function; the hyperbolic tangent model used the hyperbolic tangent function to describe the nonlinear relationship between stress and strain under large deformations. Later developments also included the time-dependent Burgers model [39] for describing the nonlinear characteristics of material viscoelastic behavior over time; the temperature-dependent thermo-mechanical large deformation response model [40,41] combining linear and nonlinear springs with dashpots, incorporating typical polymeric behavior such as shear thinning, thermal softening at higher temperatures and nonlinear dependence on deformation and loading rate; the strain-dependent Finitely Extensible Nonlinear Elastic—Peterlin (FENE-P) model [42] modified the effect of the degree of deformation (strain) on stress.

Overall, understanding how to describe the transient responses of viscoelastic fibers under external forces and their variation patterns with temperature, time, and strain is crucial for comprehending the nature of the cutting process. This process also involves describing the nature of tool motion under different processing conditions and the forces acting on the fibers, which cannot be solely explained by the materials’ viscoelastic properties alone. The accuracy of this description is key to understanding the fundamental tool-workpiece interaction and the characteristics of the cutting process.

## 3. Fiber Cutting Mechanism

The three categories of fibers mentioned above are all considered difficult-to-cut fibers. However, from a materials science perspective, the reasons for their machining difficulty vary. In the field of traditional machining, we typically use the term “machinability” to describe the difficulty level with which a material is processed. Machinability is generally evaluated in terms of cutting forces, surface quality, and tool wear. When applying this concept to fiber cutting, we tend to prefer using “cuttability” to describe it. The following sections will continue to analyze how cuttability, from a materials science perspective, affects the tool and machining quality.

### 3.1. Statics, Kinematics, and Dynamics Analysis of the Fiber Cutting Process

The force state of fibers during cutting is a primary factor influencing the fiber fracture mode. Analyzing the forces on fibers during cutting is not inherently difficult, but approaching the analysis by simply treating fibers as rigid bodies is not conducive to understanding the cutting mechanisms of non-brittle materials. Therefore, the difficulty in analyzing the force state of large-deformation fibers mainly lies in defining the elastoplastic state and deformation characteristics of different fiber types, as well as in combining the materials’ own stress–strain characteristics with the force relationship.

(1) Fibers in free form

Cutting fibers in a free form refers to a situation where the fibers are not fully positioned and clamped and are typically only fixed at both ends or subjected to a certain pre-tension. The main factors affecting the force state of the fibers during cutting include the tool movement pattern, tool angle, fiber tensioning method, presence of support, and the hardness and strength of the support material.

**Figure 2:**
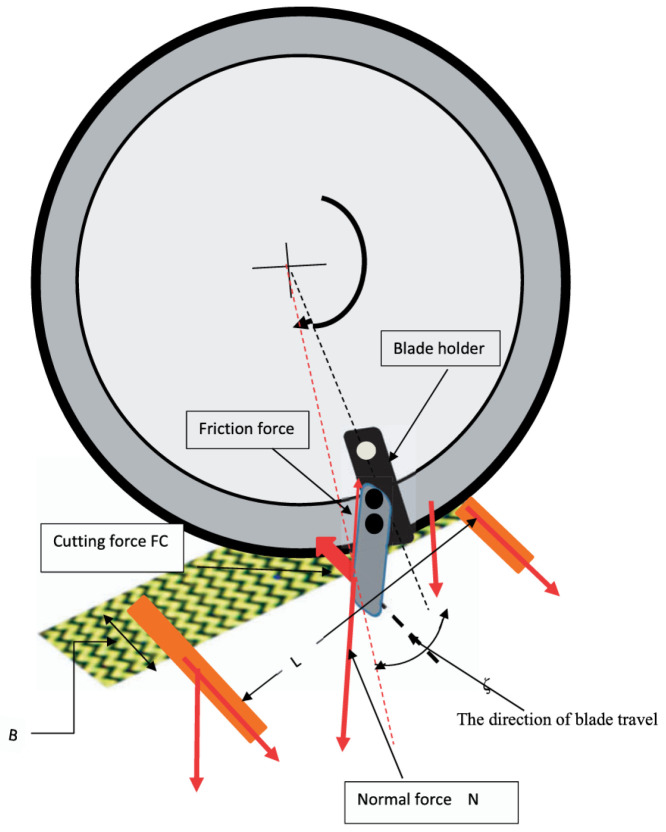
Filament yarn shear mechanism [43].

**Figure 3:**
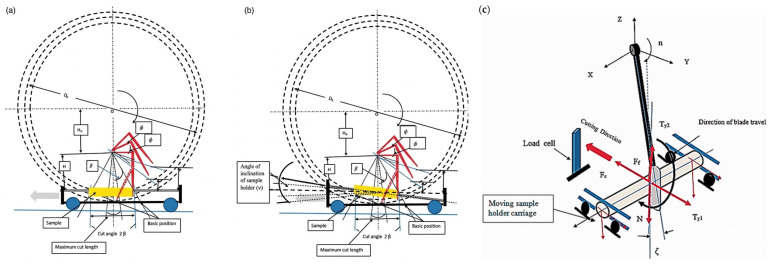
Movement of the cutting blade through the fiber yarn. (**a**,**b**) Trajectory of the cutting blade edge during rotation of the blade holder; (**c**) mechanics of cutting [44].

**Figure 4:**
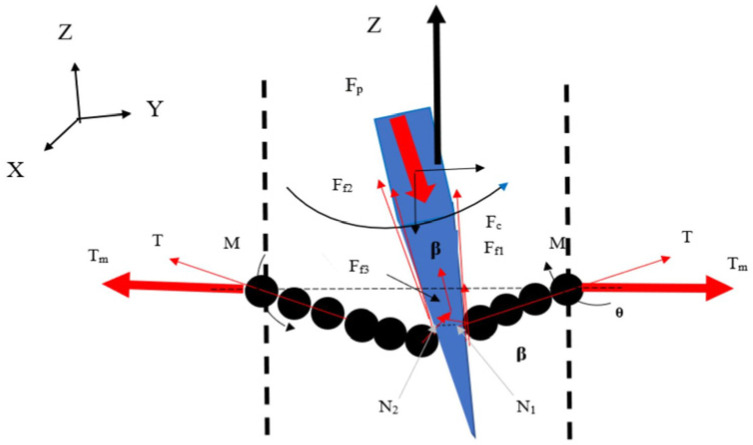
Forces acting on the blade during the slashing process [45]. *F*_p_: slashing force. *F*_c_: yarn-cutting force. *F*_f1_: friction force between the blade and the contacted yarn. *F*_f2_: friction force between the blade surface and the contacted yarn. *T*: tension on the fabric due to slashing. *N*_1_, *N*_2_, and *N*_3_: normal forces due to the pressing of the yarns on the blade surface during slashing. *M*: moment due to the instantaneous deformation of the fabric under the slashing. *b*_i_: instantaneous width of the blade.

Installing a number of evenly distributed blades on the circular cutter can effectively improve cutting efficiency. The model in Figure 2 simulates cutting in the case of stabbing or slashing the fiber textile, which depends on the blade cutting angle, blade sharpness, coefficient of friction, fiber material, the value of normal force on the sample, cutting angle, and cutting speed [43]. The fibers were installed at both ends and pre-tensioned using a static weight, and the changes in equivalent stress and yarn shear stress were analyzed through the deformation of the fibers during cutting, with the movement of the cutting blade through the fiber yarn illustrated in Figure 3 [44]. When the blade on the circular cutter slashed onto the fabric, the fabric experienced tensile forces, shear forces, and compression forces that can affect its structure and properties [45]. During normal cutting of the angle *θ* = 0, the value of N equals T, and M equals 0. However, when the blade is inclined, a component of the forces pushes the yarn to slide along the blade edge. The relative position of the yarns on the edge of the blade is determined by the physical properties of the fabric and its design, as shown in Figure 4. The higher the fabric stiffness, the higher the value of M, and the more difficult the fabric is to slash.

**Figure 5:**
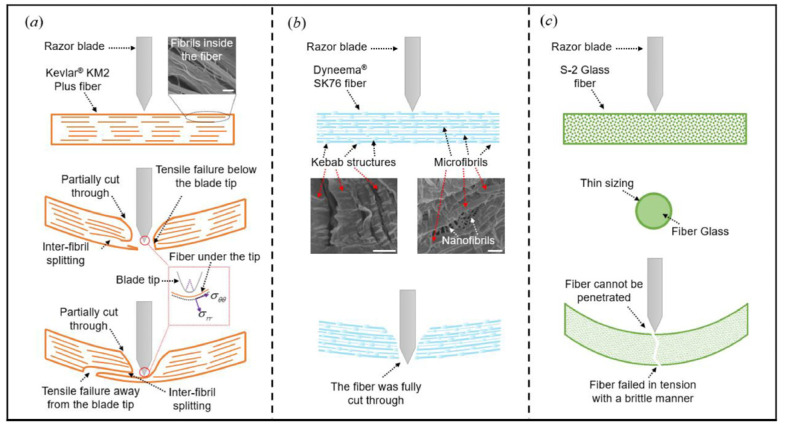
Failure mechanisms of different fibers under dynamic normal cutting. (**a**) Kevlar^®^ KM2 Plus (DuPont, Wilmington, DE, USA); (**b**) Dyneema^®^ SK76 (DSM Dyneema, Urmond, The Netherlands); (**c**) S-2 Glass (AGY Holding Corp., Aiken, SC, USA). Scale bar is 500 nm [49].

The cutting angle is a controllable factor that alters the force state of the fiber. JB Mayo Jr. investigated the effect of cutting angle on the cut resistance of different high-performance fibers, finding that both organic and inorganic fibers showed less cut resistance as the cutting angle was increased [46]. HS Shin found that the cut resistance of all fibers depended strongly on slice angle [47]; both cut energy and failure initiation strain decreased with decreasing slice angle, blade sharpness, and yarn pre-tension [48]. Jinling Gao [49] reviewed that an increasing cut angle was the possible reason for the decline in the dissipated energy and illustrated the failure mechanisms of different fibers under dynamic normal cutting, as shown in Figure 5.

**Figure 6:**
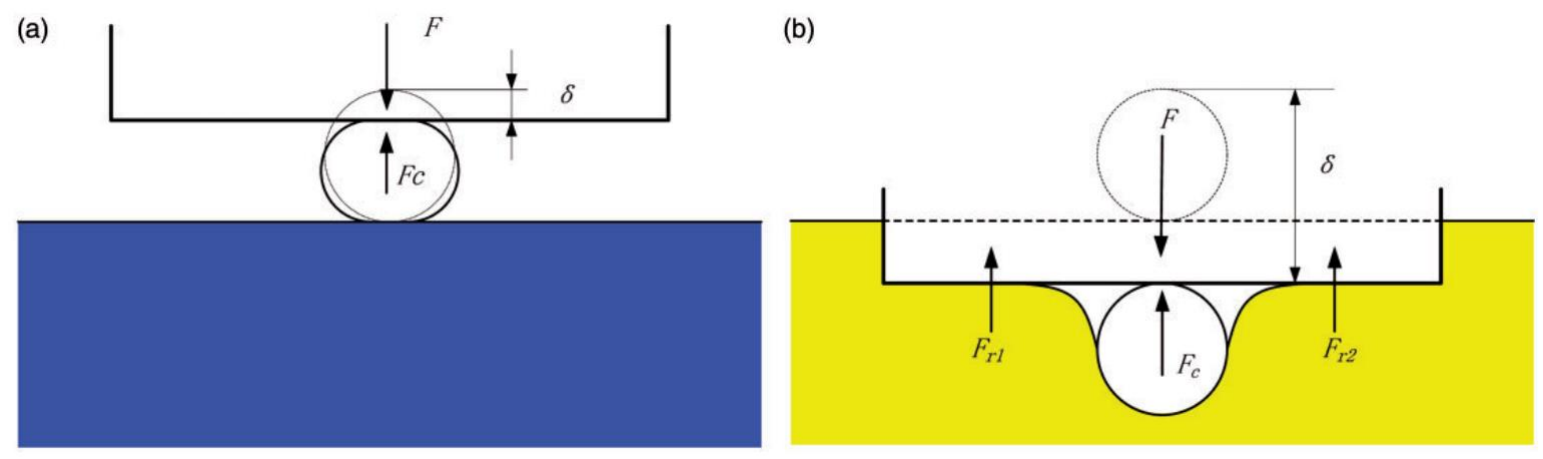
Schematic of a single carbon fiber contact with the fixing constraint [50]. (**a**) rigid-fixing cutting; (**b**) flexible-fixing cutting.

During the cutting process, the degree of deformation of the support is closely related to the degree of deformation of the fiber. When cutting brittle fibers with a soft support, it can be simplified as the support deforming while the fiber remains undeformed; with a hard support, it can be simplified as the support remaining undeformed while the fiber deforms [50]. Figure 6 shows the difference in the cutting of brittle fibers under soft and hard support. The chopping process of a carbon fiber bundle was not a simple superposition of every single monofilament fracture, in which the rigid support caused the brittle fibers at the edge of the bundle to slide to the lower layer, and the fiber slippage phenomena and intermediate fracture behaviors made the deformation and stress of the fibers more complicated [51]. Furthermore, there is a significant difference in the effect of the force state and deformation on fibers between supported and unsupported cutting. The failure of the single carbon fiber was caused by the tensile effect in an unsupported cutting, whereas it was caused by the bending effect in a flexible-supported cutting [52]. For cutting a large quantity of deformable UHMWPE fibers, quantifying the expansion (with elastic support) or elongation (without support) of the fiber fracture surface can be the main indicator of plastic deformation during the cutting process [53].

(2) Fibers in fixed form

When fibers are embedded in a prepreg or matrix at different lengths and angles to become part of a composite material, their force state is in a fixed form.

**Figure 7:**
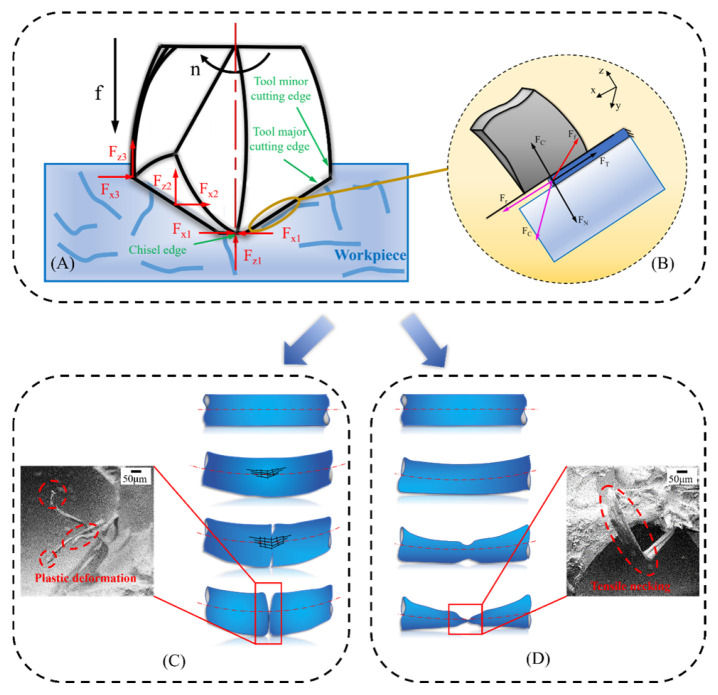
(**A**) Analysis of the force of short fiber-reinforced material; (**B**) analysis of the force on the fiber left in the hole wall; (**C**) extrusion > tension; (**D**) tension > extrusion [54].

Based on the length of the fibers, there are two categories in this form. The first type is short fibers evenly scattered in the matrix. The force state of short fibers is variable depending on the conditions. They are observed only when necessary, during the evaluation of composite processing quality. The influencing factors include the friction coefficient of the fiber surface, the wettability between the fiber and the matrix, the cuttability of the fiber itself, the force state of the fiber, and the processing methods, etc. Chaolong Fu [54] found that two different mechanics, tension > extrusion and extrusion > tension, decided the breaking mode of short fibers in the composite in different ways. As shown in Figure 7, when extrusion > tension, the short fibers in composite materials undergo shear fracture, while tensile fracture occurs when tension > extrusion. M. Khafidh [55] discussed crack propagation in silica and short-cut aramid fiber-reinforced elastomers and found that the fibers aligned parallel to the sliding surface were pulled out.

**Figure 8:**
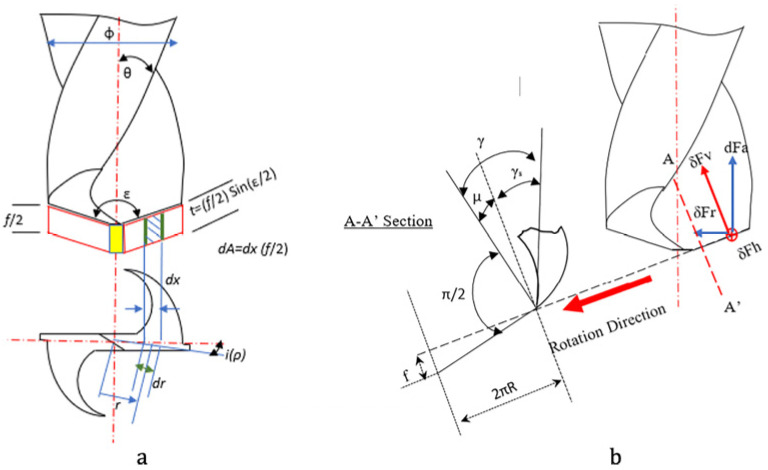
Chip load and cutting forces at the cutting lip of long fiber reinforced composites [57]. (**a**) Chip load present on the element at cutting lip. (**b**) Representation of cutting forces at the cutting lip with geometric parameters such as rake angle.

The second type is long fibers embedded in the matrix with a fixed arrangement and orientation. In addition to the factors mentioned above for scattered fiber-reinforced composites, the influencing factors for long fibers are also closely related to fiber orientations. Extensive kinematic and static/dynamic analyses have been conducted on the effect of fiber orientation, ranging from initial analyses of cutting types for different fiber orientations [56] to assessments of chip load in conventional/inclined drilling and the interaction between the drill bit and fiber orientation [57] (Figure 8) and numerical and simulation modeling of the impact of fiber orientation on cutting [58,59]. Dong-Gyu Kim [60] noticed the issue of fiber deflections in composites and then modeled the fiber and matrix as a beam to discuss the interdependent relationship between cutting force, fiber deflection, the proportion of uncut fibers, and surface roughness.

Overall, the cutting behavior of both short and long fibers in non-free configurations is governed by fiber-matrix interface interactions and processing methods. However, there are significant differences between these two areas of research. As short fibers are randomly distributed and subjected to random stresses, research has largely centered on observing their failure modes; whereas, as long fibers are fixed in their arrangement, fiber orientation becomes a key influencing variable.

### 3.2. Fibers Cutting Facture Mechanism

The foregoing discussion reveals that the fracture patterns of cutting fibers exhibit significant variation depending on their distinct kinematic and dynamic states.

(1)  Fibers in free form

**Fiber types:** Three typical cut-induced fiber failure modes for organic fiber (Kevlar^®^ KM2 Plus fiber), UHMWPE fiber (Dyneema^®^ SK76 fiber), and brittle fiber (S-2 Glass fiber) were partially cut through, followed by tensile failure, being fully cut through, and tensile failure without penetration [49]. However, high-performance fiber cutting is always full of problems and difficulties; it should clearly distinguish between damage caused by cutting and tearing. For Kevlar fiber slashing experiments, microfilaments were pulled from the core of the filament and split to form the fibrillation phenomenon during [45]. For jute fiber bundle cutting, there existed an obvious compression process before rupture in a quasi-static cutting, and the cutting force had a tendency to turn into an impact when the blade speed was too fast [61]. For carbon fiber cutting, the cutting-off mode showed different conditions due to various geometrical positions, which included compressional fracture, bending fracture, and shear fracture [62].

The loading rate also plays a crucial role in the fracture behavior of brittle fibers. Experimental studies on SiC, glass, and carbon fibers have shown that their fracture morphology and strength are strain-rate dependent. At higher dynamic loading rates, the dominant fracture mode can shift, which directly correlates with changes in the measured fiber strength [63]. This sensitivity necessitates careful consideration of the operational environment when designing components with brittle fiber reinforcements.

**Figure 9:**
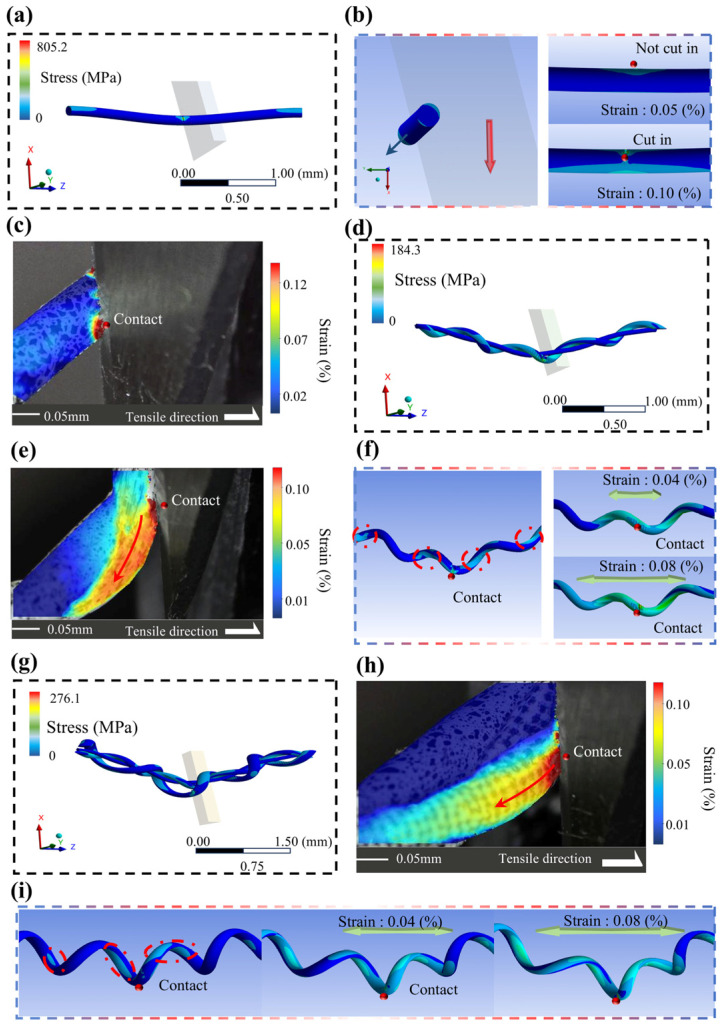
Cut-resistant simulation results of UHMWPE, wire, W-H/MCY, and DW-H/MCY materials. (**a**) Cutting simulation result of the UHMWPE and wire; (**b**) local effects of cutting simulation of UHMWPE and wire; (**c**) cutting DIC result of the UHMWPE and wire; (**d**) cutting simulation result of W-H/MCY materials; (**e**) cutting DIC result of W-H/MCY materials; (**f**) local effects of cutting simulation of W-H/MCY materials; (**g**) cutting simulation result of DW-H/MCY materials; (**h**) cutting DIC result of DW-H/MCY materials; (**i**) local effects of cutting simulation of DW-H/MCY materials [64]. The dashed red circles indicate the various stress concentration points. The arrow indicates the direction of decreasing stress.

**Fiber braided structures:** The cutting characteristics of fiber braided structures undergo significant changes. When cutting the fiber/wire composite yarns integrated with high-performance fibers and metal wires (H/MCY), the bearing/shear stress applied to this fiber braided structure was distributed into multiple segments and propagated to other parts of the structure, and the simulated results in Figure 9 confirm the distribution of the bearing/shear stress of H/MCY [64].

**Figure 10:**
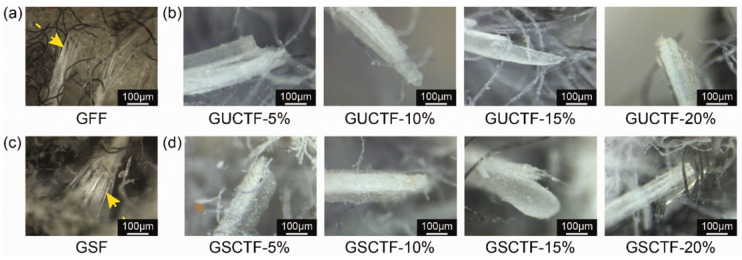
Failure morphology of (**a**) GF fabric; (**b**) GUCT fabrics with different HEMA concentrations; (**c**) GS fabric; (**d**) GSCT fabrics with different HEMA concentrations [65]. GF and GS fabrics exhibit glass fibers (indicated by yellow arrows) exposed in a radial pattern on the surface, which compromises wearing comfort (**a**,**c**).

**Cut resistance:** In order to evaluate the performance of emerging novel fibers, numerous studies observed their fracture characteristics during cutting by focusing on the cut resistance properties of the fibers [46,47]. For instance, when the outer layer of glass fiber was coated with polyurethane acrylate (PUA) resin, the fracture behavior of different fabrics, as shown in Figure 10, changed from a large axial fracture range to more fiber aggregation at the fracture point and less fiber pull-out [65]. Lijuan Wang applied the rheological theory of fabric to evaluate the stress–strain relationship of UHMWPE yarn and to interpret the yarn cut failure mechanisms by the yarn dynamic responses and the contact volume with blades [66]. The difference in the cutting resistance of the organic fibers may be directly related to the molecular structure, which characterizes the orientation of the chains and bonding of the polymer molecules [43]. However, these studies have only focused on improving the performance of fibers without considering the tool performance and lifespan during the cutting process, and they have rarely discussed the interaction between the cutting tools and fibers.

(2) Fibers in fixed form

As reinforcing additives in composite materials, fibers in a fixed form were extensively studied. This research only summarized the cutting/fracture mechanism of fibers in this state.

**Figure 11:**
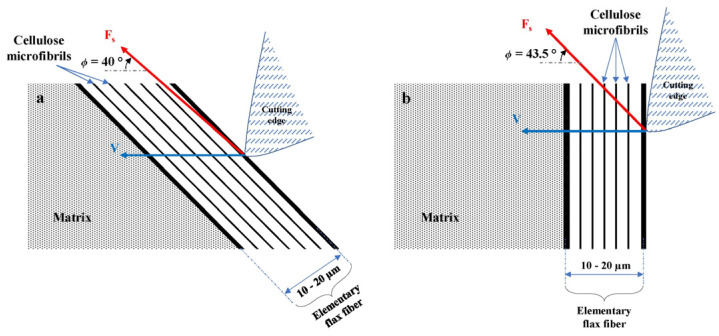
Schematic illustration of the microscopic contact between the cutting tool edge and the flax fiber. (**a**) at θ = 45° and (**b**) at θ = 90° [69].

**Fiber orientation:** Fiber orientation relative to the cutting direction is consistently reported as the most influential factor on cutting force. Cutting parallel (0°/180°) or perpendicular (90°) to fibers generally requires less force than oblique angles (e.g., 45°, 135°) [67,68]. Oblique orientations also increased surface roughness and subsurface damage, which often resulted in higher forces due to increased bending and shearing components. However, some natural fiber-reinforced composites exhibited the best machinability at a fiber orientation of 45° (Figure 11) [69]. This occurs because natural fibers typically exhibit low axial stiffness, but their lateral flexibility and interfacial bond strength with the polymer matrix are relatively low [70,71]. Consequently, when a natural fiber was contacted with a 45°–angled cutting tool, it first underwent bending deformation, followed by fracture under tensile stress. This bending–tensile fracture mode generally required a lower energy input compared with brittle fracture at the 0° direction or shear fracture at the 90° direction. Furthermore, compared with other angles, the failure mode at 45° may result in less macroscopic damage, such as fiber pull-out, matrix tearing, and delamination, thereby yielding better surface quality and lower subsurface damage [69].

**Figure 12:**
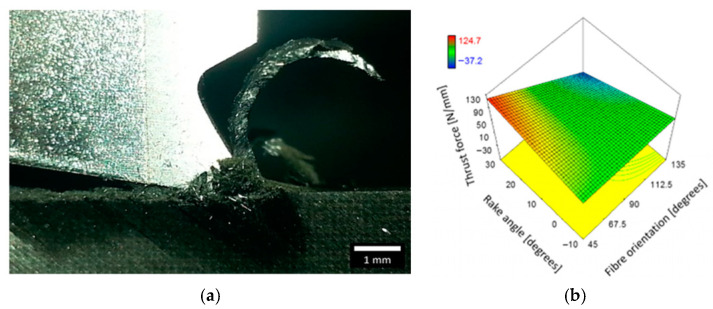
Combined effect of depth of cut and tool rake angle on the thrust force for samples with fiber orientation 90° [73]. (**a**) Chip formation for experiment 34 at a fiber orientation of 135°, a rake angle of 30°, and a cut depth of 150 µm. (**b**) Combined effect of fiber orientation and tool rake angle on the thrust force when machining at DOC: 100 µm.

**Process conditions:** The anisotropic and heterogeneous nature of fiber-based materials means that even small changes in tool geometry and process parameters can lead to significant differences in the cutting mechanism and surface quality. Higher positive rake angles generally reduce the cutting force by transforming the chip formation mode faster [72] and promoting efficient shearing rather than crushing or bending [73], as observed in Figure 12, the impact of different combinations of the depth of cut and rake angle on the thrust force. Cutting speed had a lesser but still notable effect—higher speeds can reduce forces up to a point [74] but may increase tool wear under the effect of thermal and mechanical coupling [75]. The feed rate was often the most significant machining parameter after orientation; higher feed rates increased both force and surface roughness [74]. Depth of cut played a vital role in the principal cutting force [76]. The study examined the effects of processing parameters on cutting fibers in a fixed form from different angles, providing valuable insight into how fibers are cut in composites.

**Fiber types:** Fiber types definitely influence their fracture mechanism. High-modulus fibers such as Kevlar require greater specific cutting forces than carbon fibers because of their fracture characteristics [45]. Comparing short fibers, longer fibers, or higher volume fractions dramatically increased cutting resistance [74].

**Interfacial relationship:** Friction and the interfacial relationship between fibers and tools provide a microscopic explanation for fiber fracture. When the melting resin was unable to support the fiber, weak interfaces between resin and fiber promoted debonding/pull-out rather than clean cuts [77]. In micromechanical finite element (FE) models, cutting forces were almost insensitive to micro-friction between cutting tools and composite phases (flax fibers and PLA matrix), but thrust forces were highly sensitive [57]. Higher friction at the fiber–tool interface increased cutting/thrust forces [57], specific cutting energy [78], and contact temperature, promoting delamination, uncut fibers, fiber pull-out, and burrs, especially in abrasive carbon/glass fiber composites [79,80]. During the cutting process, friction between the cutting tool and the workpiece, together with the plastic deformation of the material, generated a significant amount of heat, resulting in a marked temperature gradient in the cutting zone [81]. When the local temperature rose and exceeded the glass transition temperature of the polymer matrix, the strength and stiffness of the matrix declined sharply; this softening effect directly affected the bond strength between the fibers and the matrix, thereby increasing the likelihood of inter-fiber delamination and fiber pull-out [82,83].

Table 2 systematically summarises the cutting and fracture mechanisms for different fibres under various conditions.

### 3.3. Condition of Fiber Cutting Tools During Processing

Since changes in cutting tools during composite materials processing are caused not only by the fibers but also significantly by the matrix, it is beyond the scope of this discussion.

Current research on long, thin blades for cutting fibers mainly involves the wear characteristics of cutting tools, blade shape, and its geometric characteristics, and the effect of tool materials.

**Figure 13:**
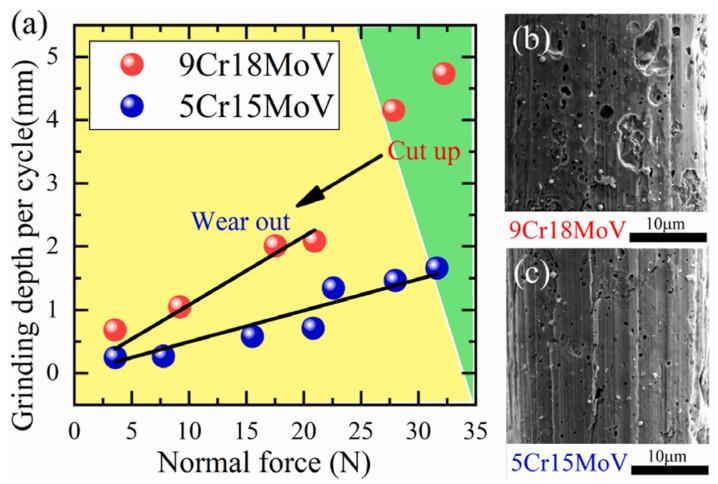
Compare wear-out depth of paper cards (**a**) by steel blades, rougher tip surface of (**b**) 9Cr18MoV than (**c**) 5Cr15MoV may result from its higher carbide content and cause a higher wear-out rate [86].

**Wear characteristics:** Crescent shapes were formed along the tool edge at several cutting positions, which were due to an uneven wear volume caused by the uneven distribution of filaments during whirling. The tool wear pattern from chopping carbon fiber is a combination of rake face wear (rapid wear stage) and cutting edge rounding (CER) (steady wear stage) [84,85]. Dong Wu discussed the relation between sharpness retention and the wear of knife blades and found that the cutting mechanism shifted from cutting to wear-out as the contact pressure underneath the blade tip fell below a critical value (Figure 13) [86].

**Figure 14:**
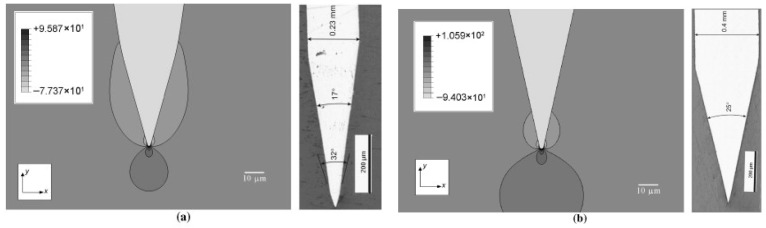
Stress in the x-direction, σ_xx_, at 2 mm blade indentation (**a**) CAMB blade (maximum tensile stress = 95.87 MPa) (manufactured by CAMB Machine Knives International (type CMK 152)), (**b**) SM blade (maximum tensile stress = 105.9 MPa) (manufactured by Swann-Morton) [88].

**Blade shape/geometry characteristics:** With the blunter blade, the cut force of yarn was higher, the initiation of yarn breakage was delayed, and yarn displacement along the blade was longer [66]. JB Mayo Jr. [46] found that the action of a very dull blade induced tension in the fibers, which failed in tension at an axial stress value that scaled with fiber diameter. Hyung-Seop Shin [48] tested the blade sharpness and found that a blunt blade would delay the initiation of fiber breakage. At microscopic scales, the blade geometry characteristics are critical. Gabriele Greco proposed a new bionic blade to imitate the micro-serration on spider fangs, and mechanically cut tough fibers, and proved the cutting efficiency of micro-serration structures on blades [87]. Different blade geometric variables have an influence on the sharpness of a blade, with the blade sharpness index (BSI) being most sensitive to tip radius; the wedge angle of 32° for the double wedge angle CAMB blade had the maximum stress approximately 10% higher than the wedge angle of 25° for the single wedge angle SM blade, as shown in Figure 14 [88].

**Tool materials:** A ceramic blade, by virtue of its high hardness, is not expected to blunt as readily as a metallic blade [48]. While ceramic tools possess high hardness and thermal stability, their brittleness can limit performance in interrupted cuts, potentially leading to catastrophic chipping, especially at very sharp edge radii [89]. Advanced materials, such as specific cermet tool materials (e.g., Ti(C_7_,N_3_)/TiB_2_/WC), have been designed based on wear prediction models to optimize their microstructure for enhanced performance [90]. The Young’s moduli and alloying elements of a blade also play key roles. Higher Young’s moduli gave lower cutting stresses on the blade edge while cutting organic and inorganic fibers [43]. 154CM steels with higher Mo content demonstrated superior capability in sharpness retention to 440C and N690 steels, although these steels exhibit similar chemical composition, carbide phases, microstructure, and HRC hardness [91].

**Figure 15:**
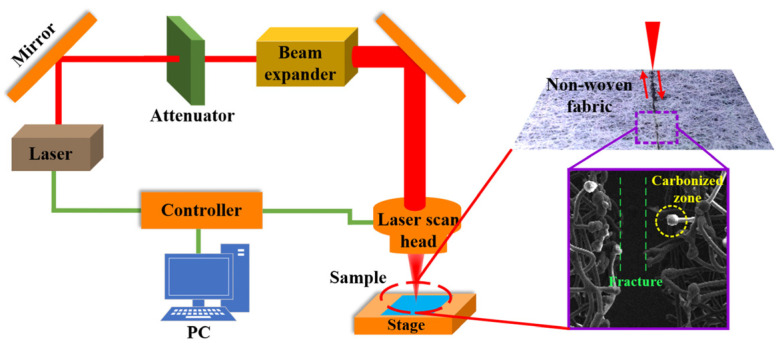
Process schematic for laser cutting of non-woven fabric [97]. The arrow indicates the path of the laser beam.

**Non-contact methods for cutting fibers:** Furthermore, non-contact methods for cutting fibers (such as laser [92,93], waterjet [94,95], ultraviolet light [96,97] cutting, etc.) have gradually become available. In these methods, the cutting tools and fibers do not come into direct contact, and their interaction mechanisms are significantly different from those of contact-based processing. Among these non-contact technologies, laser cutting stands out as the most extensively developed. Figure 15 demonstrates the laser cutting process of non-woven fabric [97]. The process relies on the focused delivery of photonic energy to induce material removal through thermal mechanisms such as melting, vaporization, and pyrolysis, or through photochemical bond-breaking in the case of high-energy ultraviolet photons [98]. Research has shown that lasers of different wavelengths have different effects on the quality of CFRP processing. In terms of CFRP cutting, continuous-wave (CW) lasers were more efficient but generated greater thermal effects, whereas pulsed lasers can reduce the heat-affected zone [99,100]. Although laser processing involves no tool wear or cutting forces, it may lead to ablation of the resin matrix and the heat-affected zone, while waterjet cutting is environmentally friendly but may cause defects in the material [101]. Water-jet cutting, particularly abrasive water-jet (AWJ) cutting, employs high-velocity fluid dynamics and particle impact to break apart the materials. This method is effective for thick, multi-layered composites. However, improper parameters such as pressure, traverse speed, or abrasive type and size can lead to fiber pull-out or matrix erosion [102]. Unfortunately, systematic research into the application of these methods in the field of fiber cutting has yet to be conducted.

In short, research on fiber cutting tools is relatively conventional and far behind current industrial production demands. Unlike previous reviews, which have primarily focused on individual fibers or emphasized the processing of composite materials rather than the fibers themselves [103,104], this review summarizes the cutting mechanisms of both natural and artificial fibers and highlights research gaps in this area. For example, when cutting viscoelastic materials, what are the effects of interfacial properties such as frictional heat, tool surface coatings, and surface micro-textures on the viscoelastic transformation of the material? What is the relationship between material characteristics, tool wear, and chip adhesion, and how do these factors influence the tool failure process? The difficulties in these questions can be summarized into two aspects. First, during the cutting process, the fibers are in a free form without complete positioning and clamping. Combined with their highly deformable characteristics, this results in a complex motion mechanism of “the tool moves, and the fiber also moves,” making it difficult to accurately describe the kinematic and dynamic relationships between the fibers and the cutting tools. Secondly, the cutting and fracture characteristics of artificial and natural fibers differ from those of conventional solid materials. Due to the influence of tensile and shear stress during cutting, the microscopic molecular chains of organic fibers may undergo disentanglement, covalent bond breakage, and functional group changes. Natural fibers may experience cellulose interlocking and molecular bond breaking. These effects can even occur in areas far from the tool–fiber contact zone, called feature change propagation, leading to large regional changes in material properties and consequent changes in cutting characteristics. Therefore, these two difficulties result from the interaction between the macroscopic behavior of the tools and the microscopic behavior of the fiber material structure. A methodology-based tool condition analysis for identifying the cross-scale relationships between tools and fibers should be established.

## 4. Conclusions

This paper conducts a literature analysis of the cutting mechanisms of industrial artificial and natural fibers. Compared with the machining mechanisms of various fiber-reinforced composites, research on the cutting mechanisms of different types of fibers—including force analysis, fiber fracture mechanisms, and the conditions of fiber cutting tools during processing—is far less in-depth. Existing studies have either focused on individual fibers or emphasized the processing of composite materials rather than the fibers themselves.

The reasons are twofold. On the one hand, early industrial fibers were not difficult to process; on the other hand, the wide variety and broad application of fiber composites in recent years have generated numerous manufacturing demands and research gaps, which have driven a deeper investigation. However, with the dramatic improvement in the properties of industrial fibers, significant challenges arise in cutting processes. Current research on the interaction between fibers and cutting tools suffers from significant shortcomings, including: (1) an insufficient description of the complex motion coupling mechanisms between fibers and cutting tools under free-form conditions due to the lack of definition of the elastoplastic state and deformation characteristics of different fiber types, as well as the combination of the material’s stress–strain characteristics with the force relationship; (2) the absence of a unified theoretical framework linking microscopic fiber fracture to macroscopic cutting performance and an in-depth study into the nonlinear cutting response of viscoelastic fibers especially for fibers in a free form; and (3) a lack of an accurate description of the complex motion mechanism of “the tool moves, and the fiber also moves” and fiber’s feature change propagation under cutting. These gaps severely hinder the development of efficient and precision cutting technologies for industrial fibers. Therefore, this review identifies these three gaps, thereby fully highlighting its theoretical and practical value.

Starting from the stress analysis of the fiber cutting process, then the fracture mechanism of fibers during cutting, and finally the condition of fiber cutting tools, this paper proposes to consider the essential characteristics of the fiber cutting process from both macroscopic and microscopic perspectives. It is suggested that future research on fiber cutting could be carried out in the following aspects:

(1) The establishment of a multi-scale theoretical mechanical model for fiber cutting, considering material deformation, temperature, and their effects on material characteristics.
(2) The fracture characteristics of the material are determined by combining the macroscopic behavior of the cutting tools and the microscopic behavior of the fiber material structure.
(3) The occurrence, evolution, and characteristics of tool wear in fiber cutting, and tools’ properties in a deep combination with material characteristics; as well as the cross-scale relationship of the tool–fiber interface from the macroscopic perspective of interfacial mechanics and the microscopic perspective of molecular dynamics.

## Figures and Tables

**Table 1:** Artificial and natural fibers [13].

Classification	Fiber Type	Key Features	Industrial Application
Synthetic industrial fibers [13]	Nylon 6.6	High toughness, excellent elastic recovery, and abrasion resistance.	Tire cords, seat belts, conveyor belts.
	Polyester (PET)	Low moisture regain, high UV resistance, and dimensional stability.	Geotextiles, industrial filters, sewing threads.
	Polypropylene	Lowest density (floats), chemically inert, hydrophobic [14].	Marine ropes, filter cloths, carpet backing, and automotive components [2].
	Acrylic	Superior resistance to sunlight and weathering; mimics wool.	Outdoor awnings, sunshades, protective covers.
High-performance fibers	Para-Aramid	strength-to-weight ratio (5x stronger than steel), high heat resistance (carbonizes at ~450 °C without melting)	Ballistic protection, aerospace components, and cut-resistant apparel
	Carbon Fiber	Extreme stiffness (Young’s modulus up to 500 GPa) and low thermal expansion	Wind turbine blades, aircraft primary structures, and high-end automotive frames
	Glass Fiber	High electrical insulation, non-flammable, and cost-effective reinforcement	Boat hulls, storage tanks, and printed circuit boards (PCBs)
	UHMWPE	Extremely high tenacity (>35 gpd), chemically inert, and highly resistant to moisture	Deep-sea mooring lines, orthopedic implants, and armored plates.
High tensile strength natural fibers	Flax	High vibration damping, low density (1.5 g/cm^3^), excellent UV resistance.	Automotive interior panels, high-end sports equipment (rackets, skis), and composite aerospace components.
	Hemp	Exceptional durability, naturally resistant to rot and pests, and high thermal insulation.	Construction (Hempcrete), bio-composites for car doors, insulation boards, and sustainable textiles.
	Jute	Cost-effective, high moisture absorption, biodegradable, and renewable.	Geotextiles (soil stabilization), upholstery, eco-friendly packaging, and low-cost composite reinforcement.
	Ramie	Retains strength when wet, has high luster, and is resistant to bacteria and mildew.	Marine cordage, industrial canvas, filter cloths, and high-performance blended textiles.
	Kenaf	Rapid growth cycle, lightweight, absorbs high amounts of CO_2_.	Automotive acoustic panels, food packaging, and reinforced plastics for consumer electronics.

**Table 2:** Comparison of cutting and fracture mechanisms among three fiber types.

Fiber Type	Fiber in Free Form		Fiber in Fixed Form	
	Fracture Mode in Free Form	Key Cutting Influencing Factors	Fracture Feature in Fixed Form	Key Cutting Influencing Factors
Brittle fibers	Tensile/bending/shear fracture, strain-rate dependent	Cutting angle, geometrical position, strain rate	Strongly affected by fiber orientation; prone to damage at oblique angles	Fiber orientation, support stiffness, and tool–fiber friction
High-tensile natural fibers	Fibrillation, pre-fracture compression, complex bundle fracture	Feed rate, interfacial wettability	Best machinability appears at 45° orientation	Fiber orientation, tool–fiber friction
Viscoelastic fibers	Tensile/shear failure, cutting-speed dependent	Feed rate, braided structure, blade contact volume, yarn dynamic response	Significantly affected by temperature; prone to thermal softening	Temperature, tool–fiber friction

## Data Availability

The raw data supporting the conclusions of this article will be made available by the authors upon request.
